# Targeting a Conserved Lysine in the Hydrophobic Pocket of HIV-1 gp41 Improves Small Molecule Antiviral Activity

**DOI:** 10.3390/v14122703

**Published:** 2022-12-02

**Authors:** Li He, Guangyan Zhou, Vladimir Sofiyev, Eddie Garcia, Newton Nguyen, Kathy H. Li, Miriam Gochin

**Affiliations:** 1 Department of Basic Sciences, Touro University California College of Osteopathic Medicine, 1310 Club Drive, Mare Island, Vallejo, CA 94592, USA; 2 Master of Science in Medical Health Sciences, Touro University California College of Osteopathic Medicine, 1310 Club Drive, Mare Island, Vallejo, CA 94592, USA; 3 Department of Pharmaceutical Chemistry, UCSF School of Pharmacy, San Francisco, CA 94143, USA

**Keywords:** HIV-1, gp41, covalent inhibitor, fusion inhibitor

## Abstract

Human Immunodeficiency virus (HIV-1) fusion is mediated by glycoprotein-41, a protein that has not been widely exploited as a drug target. Small molecules directed at the gp41 ectodomain have proved to be poorly drug-like, having moderate efficacy, high hydrophobicity and/or high molecular weight. We recently investigated conversion of a fairly potent hydrophobic inhibitor into a covalent binder, by modifying it to react with a lysine residue on the protein. We demonstrated a 10-fold improvement in antiviral efficacy. Here, we continue this study, utilizing instead molecules with better inherent drug-like properties. Molecules possessing low to no antiviral activity as equilibrium binders were converted into µM inhibitors upon addition of an electrophilic warhead in the form of a sulfotetrafluorophenyl (STP) activated ester. We confirmed specificity for gp41 and for entry. The small size of the inhibitors described here offers an opportunity to expand their reach into neighboring pockets while retaining drug-likeness. STP esterification of equilibrium binders is a promising avenue to explore for inhibiting HIV-1 entry. Many gp41 targeting molecules studied over the years possess carboxylic acid groups which can be easily converted into the corresponding STP ester. It may be worth the effort to evaluate a library of such inhibitors as a way forward to small molecule inhibition of fusion of HIV and possibly other enveloped viruses.

## 1. Introduction

Fusion of the enveloped RNA virus HIV-1 is mediated by glycoprotein-41, part of the spike protein that decorates the outer surface of the virus. The gp41 ectodomain is a homotrimer, characterized by two heptad repeat domains NHR and CHR, separated by a loop, with a fusion peptide (FP) and transmembrane (TM) domain at the N- and C-terminal ends, respectively. During HIV fusion, the ectodomain rearranges into an antiparallel NHR-CHR six-helix bundle, resulting in close association of FP and TM and contributing to pore formation [1,2,3,4]. A deep hydrophobic pocket (HP) on the NHR is an important hotspot in stabilizing the six helix bundle [5,6]. Both the HP residues 565-LLQLTVWGIKQLQARIL-581 and the WxxWDxxI-motif in the HP binding domain on the CHR are highly conserved [7]. The HP has been a target of drugs against fusion, including pocket binding C-peptides [8] and potent D-peptides [9]. However, low molecular weight fusion inhibitors have mostly demonstrated only moderate activity against viral infectivity, typically in the low µM range [10,11,12,13,14,15].

Low molecular weight HP inhibitors typically contain multiple aromatic groups and a negative charge, conforming to a binding site with net positive charge along one edge and a large contingency of hydrophobic amino acids forming the base and opposing edge of the pocket. The most potent inhibitors have molecular weight 500 Da or larger. The failure to achieve potent fusion inhibition with small molecules may be attributed to one or both of two characteristics: (1) the nature of the pocket precludes binding by ligands that have good drug-like properties; (2) binding to the pocket in and of itself is insufficient to counteract the NHR-CHR interaction.

To probe this further, we have begun a study in which we converted equilibrium binders into covalent inhibitors. A covalent bond slows or eliminates the off-rate of the ligand, impeding or blocking the binding site [16]. Applying this strategy to HP-binding ligands would enable us to observe whether molecules with better drug-like characteristics can be obtained, and whether HP binding is sufficient to preclude fusion. In covalent inhibition, an electrophilic group on the ligand reacts with a nucleophile on the protein [17,18]. A suitable nucleophile is lysine-574 in the HP. Lysine-574 is present in almost all viral variants with the exception of an arginine substitution found in uncommon outlier groups O and P. The O group comprises up to 5% of infections in some countries in West and Central Africa, while P has been rarely observed.

**Figure 1:**
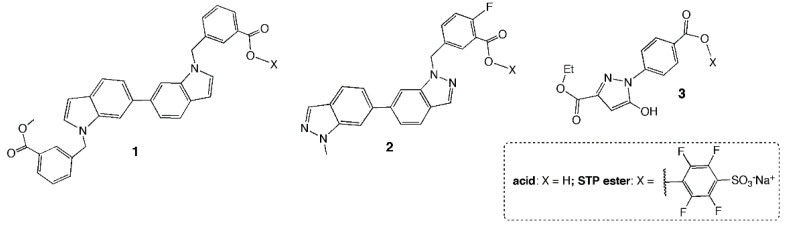
Structures of gp41 inhibitors and their corresponding sulfotetrafluorophenyl (STP) esters.

We have demonstrated the effectiveness of this strategy in a recent study, in which we used one of our most potent low molecular weight equilibrium binders, compound **1** (Figure 1). An activated sulfotetrafluorophenyl (STP) carboxylic acid ester of **1** formed a covalent adduct with lysine-574 and showed a concomitant 10-fold increase in antiviral potency with no observed toxicity [19]. STP esterification is a useful strategy for HP binders, which often contain carboxylic acid residues and are negatively charged. Furthermore, amide bond formation with lysine occurs readily, despite the high pKa of unperturbed lysine residues [20]. The approach relied on equilibrium binding potency to obtain specificity and avoid off-target effects.

Compound **1** does not have ideal properties as a drug lead, with a relatively high molecular weight (515 Da) and ClogP > 6, making it difficult to generate improved analogs. Indeed, substitution of carbon atoms with nitrogen atoms in the ring systems invariably led to a reduction in potency [14]. In the current study, we applied the strategy to smaller inhibitors, having better drug-like properties but weaker inhibitory effect. We examined whether STP esterification of these compounds could similarly lead to significantly improved potency, and whether the lower equilibrium binding affinity would still allow for selectivity of covalent attachment. Such an effect could then be amplified by extending the small molecules into adjacent pockets. Our experimental results demonstrate that the STP esters had significantly improved activity against HIV-1 fusion compared to the acid, formed a unique complex in the HP and were selective for HIV envelope.

## 2. Materials and Methods

### 2.1. Chemistry

Starting materials and solvents were purchased from commercial suppliers and used without further purification. STP esters were prepared by overnight reaction at room temperature with a stoichiometric amount of sodium 2,3,5,6-tetrafluoro-4-hydroxy benzenesulfonate and addition of dicyclohexyl carbodiimide (DCC). The resultant mixtures were separated by silica gel chromatography or extracted from methanolic solution with ether. Purity and molecular weight were determined by LC-MS and NMR. LC-MS experiments were conducted on an Agilent 1100/Bruker microTOF-Q LC-MS at the Molecular Foundry, Lawrence Berkeley National Labs. NMR experiments were conducted on a Bruker Avance 400 MHz spectrometer, with a triple resonance TXI probe for ^1^H and a QNP probe for ^19^F measurements. Details of the syntheses and analysis are provided in Supplementary Materials.

### 2.2. Binding Affinity Assay

Inhibition constants K_i_ for binding in the hydrophobic pocket were determined using a fluorescence intensity assay as previously described [21,22]. Briefly, Fe^II^(env2.0)_3_ was used to mimic the hydrophobic pocket in the gp41 NHR coiled coil. Env2.0 contains 20 hydrophobic pocket—associated residues (_565_LLQLTVWGIKQLQARILAVE_584_). It is N-terminally capped by 2,2′-bipyridine-5-carboxylate, a bidentate ferrous iron chelator that assures the trimeric structure of the NHR upon metal binding. A fluorescein (FL)-labeled pocket-binding C-peptide C18FL at 30 nM concentration was used to probe inhibitor binding. Quenching of probe fluorescence occurred in the presence of 8 µM Fe^II^(env2.0)_3_. K_I_ was determined by measuring the dose dependent fluorescence recovery in the presence of a competitive inhibitor. A modified metallopeptide construct with K_574_ replaced with norleucine was employed to measure the effect of the lysine side chain amino group on inhibitor binding affinity.

### 2.3. Protein Constructs

Protein constructs displaying the HP binding region were prepared in E. coli as previously described [23]. Molecular weight of the proteins plus adducts formed with small molecules were determined on an AB Sciex Maldi-TOF Series Explorer 7000 at the Molecular Foundry, Lawrence Berkeley National Labs.

### 2.4. Cell-Based Assays

Cell–cell fusion and virus-cell fusion experiments were conducted as previously described [19]. All cell-culture experiments were conducted in a BSL-2 laboratory. Cell lines and plasmids were obtained through the NIH HIV Reagent program, Division of AIDS, NIAID, NIH, now managed by the American Type Culture Collection. Env expression vectors were donated by C. Weiss, FDA.

Briefly, target cells used were TZM-bl cells expressing CD4, CXCR4 and CCR5 [24] and containing an integrated reporter gene for firefly luciferase under control of HIV-1 LTR [25]. For cell–cell fusion experiments, effector cells were HL2/3 which produce HXB2 Env, Tat and Rev. [26] Fusion was allowed to proceed in the presence or absence of serially diluted inhibitors for 5 h in serum-reduced medium. Luciferase expression was measured on a Biotek Synergy 2 plate reader using Luciferase Assay Reagent (Promega) according to the manufacturer’s instructions.

Virus-cell fusion experiments were conducted using pseudotyped virus to measure infectivity. Pseudotyped virus was prepared in 293T cells using Env deficient HIV-1 backbone vector pSG3Δenv [27] and Env expression vector pSM-HXB2-WTgp160, pJRFL-WTgp160 or pAmphoMLV-Env using FuGENE HD transfection reagent (Promega). A virus concentration equal to 10 * TCID_50_ was used in experiments. Target cells were exposed to virus with or without serially diluted compounds for 5 h in serum-reduced medium, after which virus and compounds were removed and the medium was replaced with DMEM complete medium. Luciferase expression was read after a further 24 h.

Control experiments included wells with vehicle only (DMSO) without compounds, and wells without virus or effector cells. Assay integrity was also regularly checked by including a titration of coreceptor inhibitor (AMD3100 or maraviroc) in control wells. Additionally, selectivity for fusion was distinguished from potential toxicity of the compounds using a resazurin assay, in which fluorescent rezafurin, produced from resazurin by the mitochondrial respiratory chain, is a marker of cell viability. This assay was conducted concomitantly with the fusion assays, since it utilizes the supernatant, while luciferase expression is contained within the adherent cells.

Full details of all cell-based experiments are provided in the Supplementary Information of reference [19].

## 3. Results

Table 1 lists the properties and observed potency of three equilibrium binders. Structures are shown in Figure 1. Compound **2** is a truncated form of **1**, with a benzoic acid ester moiety removed and two nitrogen substitutions at the 2-positions of each indole ring. Compound **3** is a fragment-like molecule with high aqueous solubility. The inhibition constant K_I_ for equilibrium binding to the HP was determined in a fluorescence assay measuring displacement of a fluorescently labeled HP-binding peptide (see Methods). All of the inhibitors bound to the HP with a K_I_ in the low µM to 200 µM range. In keeping with the nature of HP inhibition, reduction in inhibitor hydrophobicity and log P was accompanied by a higher inhibition constant for binding and reduction in antiviral potency. No measurable antiviral potency was observed for **3**.

### 3.1. STP Esters Readily Reacted with Free Lysine-ε-NH_2_

The STP ester of each compound was prepared and confirmed by NMR and LC-MS.

Reactivity of the STP ester of **1** and **2** was tested in a reaction with N-acetyl-lysine, and monitored using HPLC. **1**-STP reactivity has been previously documented [19]. **2**-STP was highly reactive, with almost complete reaction occurring after 1.5 h (Figure S1). **3**-STP reactivity was not evaluated by HPLC because of the low absorbance of starting material and product at any measurable wavelength.

**Figure 2:**
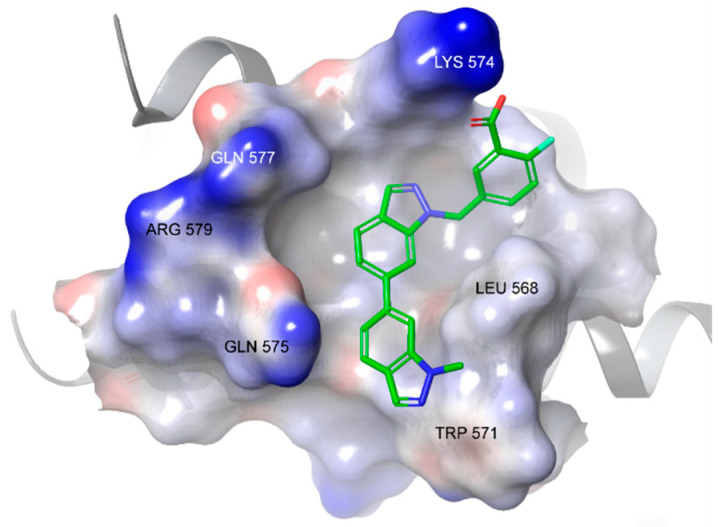
Low energy model of **2** bound in the HP, predicted by Schrodinger docking [13].

Formation of a covalent adduct with gp41 requires juxtaposition of the carboxylate group and the lysine ε-NH2 upon compound binding in the pocket. In silico docking for **1** [13,19] and **2** (Figure 2) [13] revealed just such a pose, with the hydrophobic bis-indole moiety fitting into the contours of the pocket and the carboxylic acid pointing towards the lysine. It is more difficult to predict the orientation of **3**, given its small size. Furthermore, the bulky STP ester may shift the alignment of the fragment-like ligand.

### 3.2. STP Esterification of ***2*** and ***3*** Improved Binding Affinity for the HP

**Figure 3:**
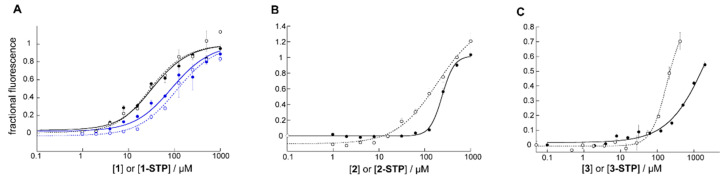
Fluorescence experiments measuring affinity of compounds by competitive inhibition. Binding curves, fit to a standard IC_50_ equation, are shown for the acid (closed circles) and ester (open circles) forms of (**A**). **1**, (**B**). **2** and (**C**). **3**. Additionally, shown in A are binding curves (in blue) to a receptor in which the pocket Lys574 had been replaced with norleucine. The fractional fluorescence was calculated relative to that of free probe peptide in the presence of compound. Data was measured repeatedly over the course of 3 h and shown at 30 min for (**A**) and 45 min for (**B**) and (**C**). Error bars show the standard deviation.

Fluorescence binding experiments (see Methods) revealed increased affinity of **2**-STP and **3**-STP for the HP, compared to their acid counterparts, by a factor of 3 and 10, respectively, as measured by the change in IC_50_ (Figure 3B,C). If approximated to an equilibrium binding curve, K_I_’s were ~10 µM for **2**-STP and ~ 20 µM for **3**-STP. In both cases, the inflection in the curve began at a concentration corresponding to the K_I_ of **2** or **3**, suggesting that covalent complex formation was predicated on equilibrium binding. The receptor used has two additional lysines apart from the pocket lysine, indicating a preference for K574 in the pocket. Interestingly, no change in the binding curve was observed by STP-esterification of **1** (Figure 3A), suggesting that the off-rate of **1** was already low. We therefore conducted a second experiment with **1**, using an alternative receptor in which Lys-574 was replaced with norleucine. The substitution leaves hydrophobic components of the interaction intact, while obviating H-bond or covalent bond formation. K_I_ increased 5 or 6 fold for the acid or ester, respectively, indicating that H-bond or covalent bond formation played an important role in the affinity of **1** or **1**-STP. With compound **2**, we observed that Nle-574 substitution increased the IC_50_ of **2**-STP by a factor of 2.2.

### 3.3. Compounds ***2***-STP and ***3***-STP Reacted with a Protein Construct of the gp41 Ectodomain

**Figure 4:**
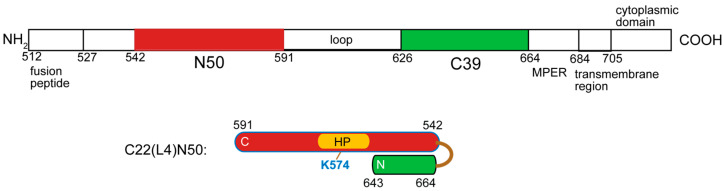
Schematic representation of the gp41 ectodomain and the hairpin construct developed to expose the hydrophobic pocket. The 705 residue ectodomain consists of the fusion peptide, fusion peptide proximal region, N-heptad repeat (N50), a loop, C-heptad repeat (C39) and membrane proximal external region (MPER). The NHR domain is shown in red and the CHR domain in green. In the hairpin construct C22(L4)N50, the 50 residue NHR is intact but the CHR is truncated by 17 residues. This, plus the placement of the CHR ahead of the NHR in the sequence, exposes the HP. Only one of the three strands of the homotrimeric structure is shown.

Gp41 ectodomain protein constructs with an exposed HP binding region were used to confirm covalent bond formation with **2**-STP and **3**-STP, as was demonstrated previously for **1**-STP [19]. Figure 4 shows a protein construct C22(L4)N50 containing a 22 residue CHR domain preceding a 50 residue NHR domain in the sequence with a short 4 residue connecting loop. Details regarding the construct will be published elsewhere. The CHR domain covers part of the NHR groove but leaves exposed the hydrophobic pocket binding region. **2**-STP and **3**-STP were stirred overnight at 37 °C with C22(L4)N50 in phosphate buffer at pH 7.8, using 100 or 300 µM protein and 0.95–1.5X compound. Maldi-TOF spectra revealed an adduct with molecular weight increase equal to the molecular weight of **2** or **3** minus a water molecule (Figure S2), as expected for amide bond formation. While adduct formation could be completed with **2**-STP, only a small amount of the adduct with **3**-STP was observed, although it was sufficient to confirm that the reaction occurred. The low yield of the adduct with **3**-STP may be related to low binding affinity of the fragment to the HP. Reactions were also verified with the construct C26(L4)N50, having a slightly longer CHR segment although still with HP exposed, and containing a second lysine at position 588, close to the end of the NHR. Similar Maldi traces were observed. The reaction of **2**-STP with the C22(L4)N50 was further evaluated by trypsin digest and LC-MS/MS, in which the mass modification site occurred predominantly at K574 (Figure S3). These experiments plus the binding experiments confirmed that the STP-activated ligands formed adducts with the lysine in the hydrophobic pocket.

### 3.4. STP Esterification Significantly Improved the Antiviral Activity of the Compounds

**Figure 5:**
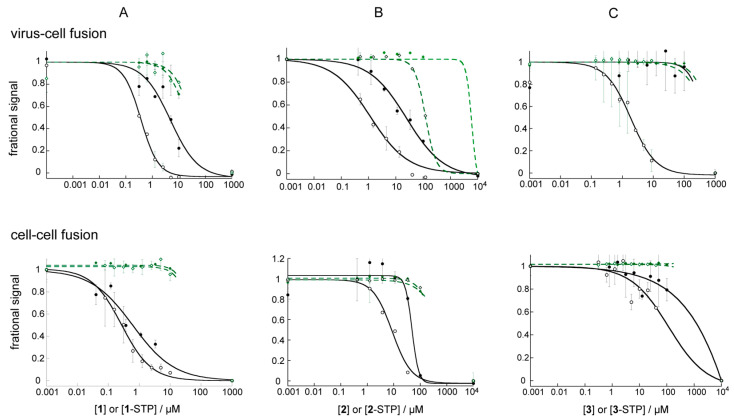
Dose response curves in antiviral infectivity and cell–cell fusion assays. Both pseudotyped virus and effector cells expressed HXB2 Env. The acid and STP ester dose response curves are overlaid in the same figure for (**A**). **1** and **1**-STP, (**B**). **2** and **2**-STP and (**C**). **3** and **3**-STP, using solid symbols for the acid and open symbols for the ester. Viability is shown in green and fusion in black. The fractional signal was normalized relative to positive and negative controls and is luminescence for the fusion readout and fluorescence for the viability readout. A fractional value of 1 indicates maximum fusion or viability. Error bars show the standard deviation.

Table 2 and Figure 5 show the results of cell-based fusion assays (see Section 2). Reproducibility was tested by repeating assays on different days, with the average of three experiments, each in duplicate or triplicate, reported in Table 2. Figure 5 shows typical dose response curves for each compound in antiviral and cell–cell fusion experiments. A fuller dataset, containing multiple repeat dose response experiments is shown in Figure S4. Cytotoxicity was measured with a resazurin assay, which measures mitochondrial output and is strongly correlated with cell health and the ability of cells to produce luciferase for the readout. We typically observed that even a 20% drop in viability as measured by this assay could interfere with luminescence results, making the fusion data unreliable at concentrations beyond this point.

It is apparent that for each of the compounds, STP-esterification improved antiviral activity significantly. The effect was greater than 10-fold for **1** (previously reported) and **3** and 7-fold for **2**. It is interesting that **3**, a small fragment with no antiviral activity, was a low µM antiviral inhibitor in the STP-ester form. One clear outcome was the increase in inhibitor selectivity, with antiviral efficacy shifting away from toxicity. This is particularly apparent for **1** and **2**, whose hydrophobic character is a potential liability [28]. Thus while 90% inhibitory concentration fell near or within the cytotoxicity window for **1** and **2**, it was well-below that in **1**-STP and **2**-STP. Slopes of the dose response curves were generally steeper for the esters than for the acids, possibly reflecting a threshold concentration above which inhibition rapidly increased with covalent bond formation. There is no evidence that STP esterification increased toxicity significantly, as might be expected for arbitrary association with lysine residues in cellular proteins.

### 3.5. The Effect of STP Esterification on Efficacy against Cell–Cell Fusion Was Not as Clear

The ability to form covalent adducts had a less clear outcome on efficacy against cell–cell fusion. A small potency enhancement upon STP-esterification was observed for **3**, from undetectable to ~120 µM, and the enhancement was about 2 fold for **1** [19]. For **2**, STP esterification converted a compound with low selectivity to one with a clear cell–cell fusion inhibitory effect and 7-fold potency improvement. The discrepancy between viral infectivity inhibition and cell–cell fusion inhibition for **3**-STP was surprising, as was the relatively high potency of **3**-STP against viral infectivity. Features that are unique to virus-cell fusion include an alternative endocytic route of entry [29,30], which does not occur in cell–cell fusion, or virus association with proteoglycans or adhesion molecules prior to CD4 engagement [30]. The difference might then be explained if the STP moiety of this small hydrophilic compound assisted with accumulation in the environment in which virus bound gp41 was unfolding. In the case of endocytic entry, however, amide bond formation would have to occur before the low pH environment of later endosomes is established, since the STP esters are reactive only near or above neutral pH (Supplementary Materials Section II).

### 3.6. STP Esters Act Specifically on HIV Env Mediated Fusion

**Figure 6:**
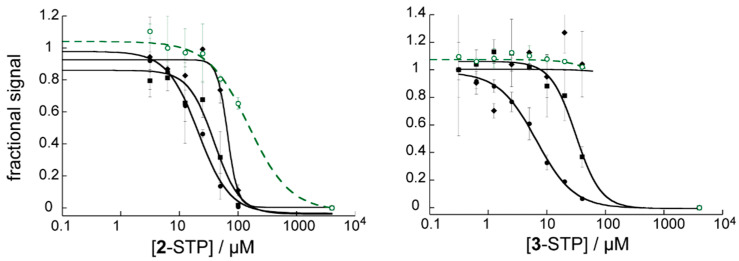
Dose response curves for compounds 2-STP and 3-STP against HXB2-Env (solid circles), JRFL-Env (solid squares) and Ampho-MLV Env (solid diamonds). Fractional cell viability compared to controls with no compound is also shown (green, open symbols). Experiment repeated in triplicate, error bars show the standard deviation. Data are fit to standard IC_50_ curves.

To confirm specificity for HIV Env and for fusion, pseudotyped virus expressing HIV-1 JRFL Env and Ampho-MLV Env were tested in dose response assays. JRFL is a faster fusing virus than HXB2 and has a lower sensitivity to fusion inhibitors [31]. Ampho-MLV fusion protein bears no relation to HIV Env. The selectivity of **1**-STP for HIV-1 Env has already been reported [19]. **2**-STP and **3**-STP demonstrated a 2- or 4-fold reduction in efficacy, respectively, against JRFL Env compared to HXB2 Env (Figure 6). The apparent activity of **2**-STP against Ampho-MLV Env was an artifact of the 40% cytotoxicity at 100 µM, with a sudden drop in luminescence readout. **3**-STP demonstrated no activity against Ampho-MLV Env pseudotyped particles.

Additional confirmation of action at the entry step was obtained from time-of-addition assays, which were used to measure the reduction in compound efficacy when compound was added 2 h after adding HXB2 pseudotyped virus to the cells. After 2 h, viral entry at 37 °C is close to complete [32]. A 14-fold increase in IC_50_ was observed for both **1**-STP and **2**-STP when compounds were added 2 h after virus. By comparison, nevirapine, a reverse transcriptase inhibitor, lost a factor of 2.7 in activity. **3**-STP was measured at a single concentration of 40 µM (5X EC_50_) and had 12% residual activity when added after 2 h. Known entry inhibitors AMD3100 (an attachment inhibitor) and T20 (a fusion inhibitor) [33] demonstrated 5% and 8% residual efficacy at 50X EC_50_ when added 2 h after virus. Acids forms of **1** and **2** showed far less discrimination than their ester counterparts, losing 2 fold activity with the delayed addition, perhaps due to non-specific off-target effects of the hydrophobic compounds.

## 4. Discussion

In this study, we examined whether conversion of gp41 HP inhibitors into covalent ligands could lead to improved efficacy of small molecules against HIV-1 fusion. Historically, gp41 HP has been refractive as a drug target to highly potent small molecule inhibitors. Specifically, we converted carboxylic acid groups into activated STP esters, which can readily form amide bonds with reactive and ligandable lysines [34]. Success with a fairly potent inhibitor, **1** in this study, has already been described [19]. We extended the study to smaller and less hydrophobic inhibitors **2** and **3**, which have lower intrinsic binding affinity to the HP, but have the potential to be expandable into adjacent crevices and pockets while retaining drug-likeness. This allowed us to test whether lower affinity ligands could still selectively target the pocket for covalent attachment, and whether this would confer sufficient improvement in efficacy to warrant additional drug design efforts against gp41.

The STP esters of **2** and **3** demonstrated 3 and 10-fold improved pocket binding affinity, respectively, over their acid counterparts, and formed adducts with proteins displaying the HP. A combination of binding experiments, Maldi and LC-MS/MS experiments strongly suggested that the principal target of covalent adduct formation was the invariant lysine-574 in the HP. This implies that the ligands adopted poses in the pocket where the carboxylate ester was in close contact with Lys-574 for proximity enabled reactivity. A large increase in efficacy against pseudoviral infectivity was observed, with EC_50_ reduced by a factor of 10 for **1**-STP, 7 for **2**-STP and > 10 for **3**-STP compared to the acids. EC_90_ differences were even larger, at 14 for **1**-STP and >10 for **2**-STP. The change for **3**-STP could not be calculated, given that **3** had no intrinsic antiviral activity up to 100 µM. Importantly, STP esterification greatly increased the discrimination between antiviral activity and toxicity, the latter compounding some measurements with **2**. There was no evidence of off-target toxicity. Off-target effects may be ameliorated by the fact that the target is extracellular, avoiding the potential consequences of intracellular destinations.

Although inhibition of cell–cell fusion was also improved by STP esterification, the effects were less clear-cut, with changes less than one order of magnitude, and weak inhibition by **3**-STP. We reasoned that the difference may lie in a possible endosomal mode of entry of virus or with the assistance of alternative attachment factors, and that the STP ester may contribute to virus-cell fusion inhibition by local concentration enhancement in the environment of the virus. Further experiments are required to explore this mechanism in detail.

We further confirmed the specificity of compounds for HIV-1 Env and fusion, by observing reduced activity in the presence of a faster fusing pseudotyped HIV virus and no activity with an unrelated pseudotyped virus displaying Ampho-MLV Env. Coupled with this was the observation of significant loss of activity in a time-of-addition assay in which viral fusion had been allowed to proceed for two hours before compounds were added to the cells. STP esterification dramatically increased the selectivity of the compounds for the fusion step.

**Figure 7:**
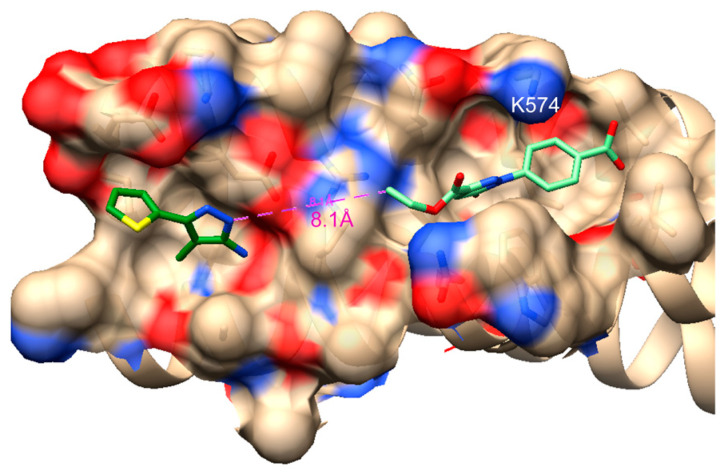
Docked pose of **3** (light green) in the HP with the carboxylate group pointing towards K574 and a previously found C-terminal pocket binding fragment (dark green).

One of the most striking findings was 9 µM inhibition of viral infectivity afforded by **3**-STP, which exceeded expectations, given that **3** is a 276 Da fragment with no activity and **3**-STP had a Ki ~ 20 µM in the HP binding assay. Molecular docking has been the main method available so far to predict how ligands bind in the HP [14,35,36], and predictions can be particularly challenging with fragment-like ligands. It is possible to use the information from this study, i.e., that that the carboxylate group should be near Lys-574, to design improved analogs. By picking such a docked pose (Figure 7), we can suggest easy modifications to **3** such as adding hydrophobic and/or amine functional groups to the benzyl ring and an electron acceptor at the ethyl ester.

The question remains whether binding in the pocket could be sufficient to inhibit the NHR-CHR interaction that occurs during HIV-1 fusion. The long protein—protein interface makes this challenging, although the concept has been proven in principle with HP binding D-peptides. They are nM inhibitors of HIV fusion, but (a) they cover a much larger footprint than a small molecule and (b) trimerization was necessary to reduce potency to sub-nM [37]. The effect of trimerization was not only enthalpic but kinetic and the low off-rate contributed to high potency. It is certainly possible to mimic the low off-rate with a covalent ligand. Additionally, enthalpic contributions could be increased by ligand expansion, not just within the pocket but to an adjacent C-terminal pocket in the same groove that has been identified as a site that contributes to the binding energetics of CHR [38,39]. We have previously identified fragments that bind specifically in this C-terminal pocket [40], and that could be tethered to a covalent HP inhibitor, and even themselves be made covalent. Figure 7 shows **3** docked in the HP and a small fragment **1a** docked in the C-terminal pocket, with a pose conforming to NMR distance constraints [40]. A distance of about 8 Å separates the two fragments, which could be bridged by a linker.

## 5. Conclusions

In conclusion, we have demonstrated that enabling proximity related reactivity with a lysine in the hydrophobic pocket of gp41 afforded on average 10-fold improvement in antiviral activity of small molecule inhibitors. Lysines are relatively abundant residues in proteins, and the technique could be extended to fusion proteins of other enveloped viruses. It is likely that the STP ester chemistry will not apply to viruses which require low pH for fusion, such as Avian Influenza virus, since the reaction is inefficient below neutral pH. Different electrophiles could be used to target conserved residues in such cases [16].

## Figures and Tables

**Table 1:** Properties of HP equilibrium binders.

Compound	MWt	Log P †	K_I_^a^	IC_50_ HXB2 ^a,b^	IC_50_ (CCF) ^a,c^
**1** *	514.57	7.38	1 ± 0.5	3	0.4
**2**	400.41	4.04	25 ± 5	35	≥50
**3**	276.24	1.10	177 ± 12	>100	>100

* published in ref [19]; † calculated using Chemaxon Marvin; ^a^ in µM, ^b^ virus—cell fusion assay using lab adapted strain HXB2; ^c^ cell–cell fusion assay. Data from cell-based assays are an average of at least three separate measurements, each in triplicate.

**Table 2:** Antiviral and cell–cell fusion assay results *.

	Antiviral Activity			Inhibition of Cell–Cell Fusion		
Compound	EC_50_	EC_90_ †	CC_50_ ‡	EC_50_	EC_90_ †	CC_50_ ‡
**1**	3.2 ± 1.6	17 ± 13	22 ± 3	0.4 ± 0.2	12 ± 6	>>10
**2**	35 ± 7	800 ± 190	≥356	78 ± 24 ^a^	110 ± 19 ^a^	≥113
**3**	>100	n/a	>100	>100	n/a	>100
**1**-STP	0.3 ± 0.2	2.9 ± 1.6	20 ± 3	0.23 ± 0.16	4.3 ± 2.8	>>10
**2**-STP	5 ± 4	59 ± 22	≥100	12 ± 3	38 ± 11	≥200
**3**-STP	9 ± 2	44 ± 15	>>40	>40	n/a	>>40

* in µM, using pseudotyped virus or effector cells expressing HXB2-Env. Results are averages of experiments repeated on different days (Figure S4), each in duplicate or triplicate. Error bars are the standard deviation. † calculated from EC_50_ and the Hill coefficient; ‡ approximated (see Figure S4); ^a^ accuracy affected by toxicity.

## Data Availability

The Los Alamos sequence database was used to examine gp41 sequences for conservation of Lys574.

## Supplementary Materials

The following supporting information can be downloaded at: https://www.mdpi.com/article/10.3390/v14122703/s1, Figures S1–S4. Chemical Synthesis [41].
