# Supplementary material for: Targeting a Conserved Lysine in the Hydrophobic Pocket of HIV-1 gp41 Improves Small Molecule Antiviral Activity
Source: Viruses. 2022 Dec 2;14(12):2703. doi: 10.3390/v14122703 (PMC9784957; doi:10.3390/v14122703)
Supplement: Supplementary file 1 [file viruses-14-02703-s001.zip › viruses-2037139-supplementary.pdf]

Targeting a conserved lysine in the hydrophobic pocket of HIV-1 gp41 improves small molecule antiviral activity

Li He [1], Guangyan Zhou [1], Vladimir Sofiyev [1], Eddie Garcia [2], Newton Nguyen [2], Kathy H. Li [3] and Miriam Gochin [1,3,*]

Supplementary Materials

Index

I.	Chemical Synthesis	p2
II.	Reaction of **2**-STP with free lysine	p4
III.	Maldi-TOF detection of protein adducts with **2** and **3**	p5
IV.	Protein site identification using LC-MS/MS	p6
V.	Repeat measurements of the compounds in fusion assays	p8

I. Chemical Synthesis

Synthesis of **2** **2** was prepared as previously described (compound **11e**) [1]

NMR ^1H ppm δ (CD_3OD): 8.11 (s, 1H), 8.01 (s, 1H), 7.93 (d of d, 1H), 7.88 (d, J=8.16Hz, 1H); 7.83 (d, J=8.66Hz, 1H), 7.81 (s, 1H), 7.74 (s, 2H), 7.56 (d, J=8.07Hz, 1H), 7.50 (d, J=8.50Hz, 1H), 7.44 (br, 1H), 7.13 (d of d, 1H), 5.73 (s, 2H), 4.14 (s, 3H). δ (acetone-d_6): 8.12 (s, 1H), 8.08 (s, 1H), 8.00 (m, 2H)7.89 (m, 2H), 7.83 (d, J=8.48Hz, 1H), 7.59 (d, 2H), 7.53 (d, 1H), 7.21 (d of d, 1H), 5.83 (s, 2H), 4.12 (s, 3H);

Synthesis of **2**-STP To a solution of **2** (40 mg, 0.1 mmol) and sodium 2,3,5,6-tetrafluoro-4-hydroxy benzenesulfonate (26.8 mg, 0.1 mmol) in anhydrous DMF (2.0 mL) was added DCC (20.6 mg, 0.1 mmol). The mixture was stirred at room temperature under N_2 overnight. TLC indicated that no starting material was left, and the mixture was dried under high vacuum to remove DMF. The crude product was purified by flash chromatography using hexane: ethyl acetate (4:3) as eluate. Afforded 35.2 mg product, white powder, yield: 53.8%.

Data for **2**-STP: White powder; MS calcd for $C_{29}H_{16}F_5N_4NaO_5S$: 650.1; found: 629.1 $(M+2H-Na)^+$. NMR: ^1H δ ppm (CD_3OD): 8.12 (s, 1H), 8.02 (s , 1H), 7.95 (m, 2H), 7.86 (d, J=8.5Hz, 1H), 7,82 (m, 2H), 7.57 (d, J-8.25Hz, 1H), 7.51 (d, J=8.03Hz, 2H), 7.15 (d of d, 1H), 5.76 (s, 2H), 4.12 (s, 3H); ^1H δ ppm (acetone-d_6): 8.16 (s, 1H), 8.13 (s, 1H), 8.04 (m, 3H), 7.94 (m, 2H), 7.88 (d, J=8.48 Hz, 1H), 7.64 (d, J=8.29 Hz, 1H), 7.58 (m, 2H),7.21 (d of d, 1H), 5.86 (s, 2H), 4.18 (s, 3H); ^{19}F δ ppm (CD_3OD) (relative to external TFA at -76.56ppm).

Synthesis of **3** Diethyloxaloacetate (470 mg, 2.5mmol) and 4-carboxyphenylhydrazine (434 mg, 2.85mmol) were dissolved in ethanol (3mL) and the mixture was heated to 80°C and stirred for 2.5 h. The product was triturated in a 1 : 1 methanol : ethyl acetate mixture to yield pure product. Yield 90%.

^1H NMR δ ppm (DMSO-d_6): 8.25 (d, 2H), 7.91 (d, 2H), 5.19 (br, 1H), 4.20 (q, 2H), 1.27 (t, 3H); δ ppm (CD_3OD): 8.05 (d, 2H), 7.95 (d, 2H), 4.33 (q, 2H), 1.37 (t, 3H)

Synthesis of **3**-STP To a solution of **3** (13.8 mg, 0.05 mmol), and sodium 2,3,5,6-tetrafluoro-4-hydroxy benzenesulfonate (13.4 mg, 0.05 mmol) in anhydrous DMF (1 mL) was added silicone gel - linked DCC (161.3 mg, 0.15 mmol). The mixture was stirred at room temperature overnight under N_2. The mixture was centrifuged at 14000 rpm for 2 minutes, the supernatant was collected and dried under high vacuum to remove DMF. Then 150 µL methanol was added to dissolve the product. The methanol solution was added dropwise with stirring to 5 ml ether. Precipitants were formed, washed twice with ether, collected and dried. A second round of methanol/ether extraction was performed. Afforded 5.3 mg product, yield: 20%. MS calcd for $C_{19}H_{11}F_4N_2O_8SNa$: 526.35; Found: 504.7 $(M+H-Na)^+$
NMR: ^1H δ ppm (CD_3OD): 8.26 (d of d, 4H), 4.33 (q, 2H), 1.38 (tm 3H); ^{19}F δ: -141.516, -155.858 (relative to external TFA at -76.56ppm)

2-STP and **3**-STP degraded over a period of months in DMSO at -10°C, but were stable in powdered form.

*II. Reaction of **2**-STP with free lysine*

6.8 µl **2**-STP (25mM in DMSO) was added to 93.2 µl N-acetyl-lysine (13.3 mM) in sodium phosphate buffer, pH 8 and incubated at 37°C for 1 – 24 hours. Aliquots were withdrawn at several time points and tested by HPLC (Figure S2). Reduction of intensity of the peak of **2**-STP at 12.8 min was accompanied by the appearance and growth of a new peak at 11.6 min. The reaction was 90% complete after 1.5 hrs (Figure S1). The reaction was also tested at pH 5.5 and was 24% complete after 3.5 hrs.

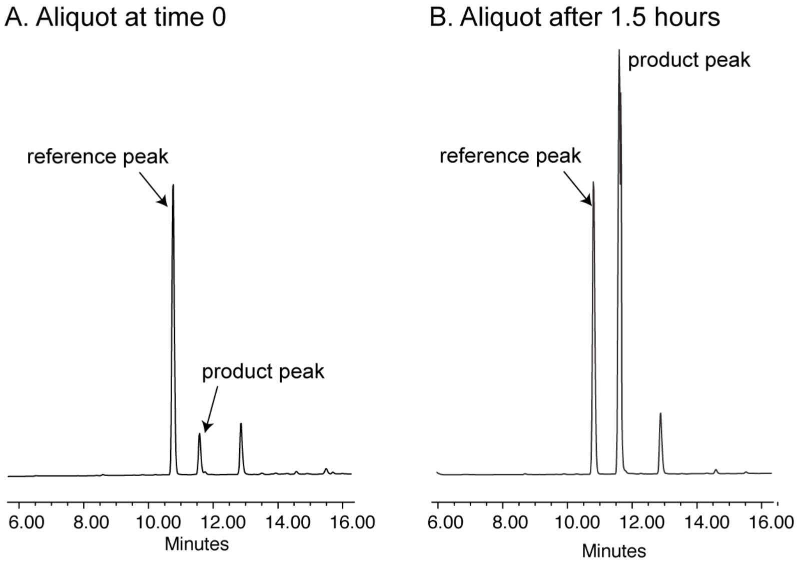

Figure S1. HPLC traces of aliquots taken from the reaction at A. time of initial mixing and B. after 1.5 hours. Intensity (A_{280}) of the product peak is 90% after 1.5 hours, when the trace is scaled to the reference peak. The reference is a small organic molecule 6-bromo-2-methyl-indazole which does not react with an STP ester.

*III. Maldi-TOF detection of protein adducts with **2** and **3***

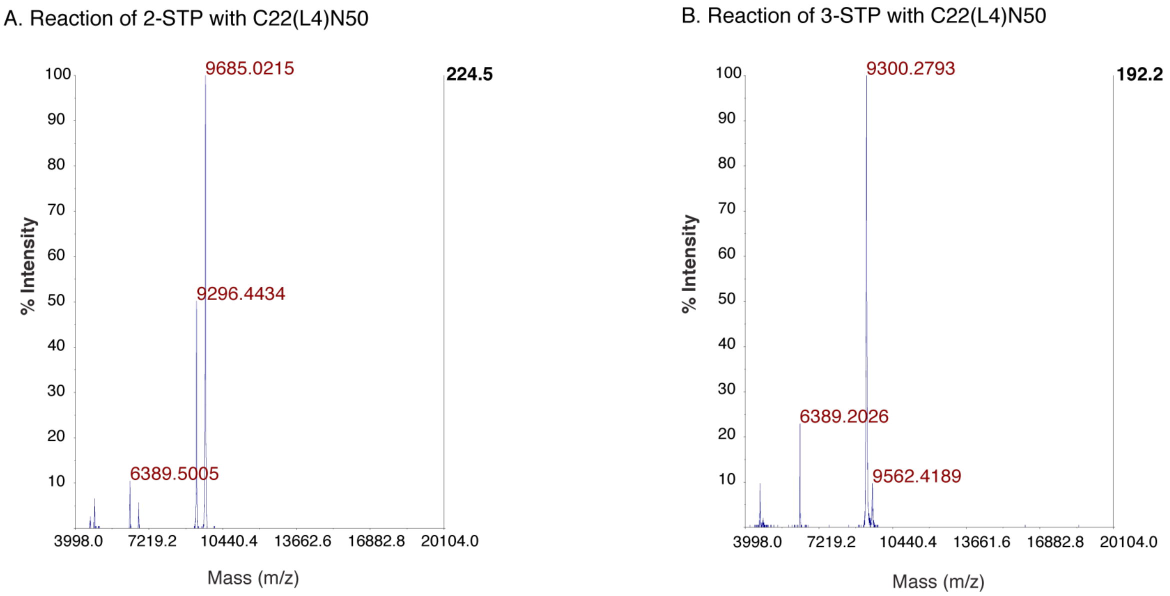

Figure S2. Maldi-TOF traces following partial reaction of A. **2**-STP and B. **3**-STP with C22(L4)N50 (9300 da). Peaks corresponding to unmodified protein and small molecule – protein adducts were observed. The observed molecular weight change was 388.6 in A. and 262 in B. With molecular weights of 400.4 da and 276.2 da for **2** and **3**, respectively, the expected changes upon covalent bond formation with loss of a water molecule are 382.1 and 258.2 respectively. The precision of the Maldi is limited to ±5 da due to the width of the peaks, as different traces and experiments verified (not shown). A small peak at 6389 da corresponds to a minor impurity in the protein sample in which the CHR has been cleaved off, but the HP is still intact. Evidence of an adduct of this segment with **2**-STP was observed.

IV. Protein site identification using LC-MS/MS

To form the covalent adduct, 2 mM **2**-STP was incubated with 300 µM C26(L4)N50 in 25mM phosphate buffer, pH 8, at 37°C for 5 hours. The protein was exchanged into formate buffer (pH 2.91) with several rounds of centrifugation using a spin filter (Amicon Ultra – 0.5ml, 3K cutoff, Sigma). 6 ug of the protein was denatured with 8M urea/100 mM ammonium bicarbonate (PH 8) where reduced with 100 mM dithiothreitol at 60°C for 30 minutes, followed by alkylation with 100 mM iodoacetamide at room temperature in dark for 1 hour. The sample was then incubated 4 hours with trypsin (1:20 weight/weight) at 37°C. The peptides formed from the digestion were further purified by C18 ZipTips (Millipore) and analyzed by online LC-MS/MS. The LC separation was carried out on a NanoAcquity UPLC system (Waters) with a linear gradient from 2 - 25% B (0.1% formic acid in acetonitrile) over 48 mins followed by 25 - 37% B over 6 mins and then 37 – 40% B over 3 mins at a flow rate of 400 nl/min. The MS/MS analysis was performed using Q Exactive Plus Orbitrap mass spectrometer (Thermo). After a survey scan, 10 most intense precursor ions were selected for subsequent fragmentation using HCD activation mode, with mass resolution of 70000 and 17500 for precursor and fragment ions, respectively. The acquired MS/MS raw data was converted into peaklists using an in-house software PAVA [2] and then analyzed using Protein Prospector search engine (http://prospector.ucsf.edu/prospector/mshome.htm).

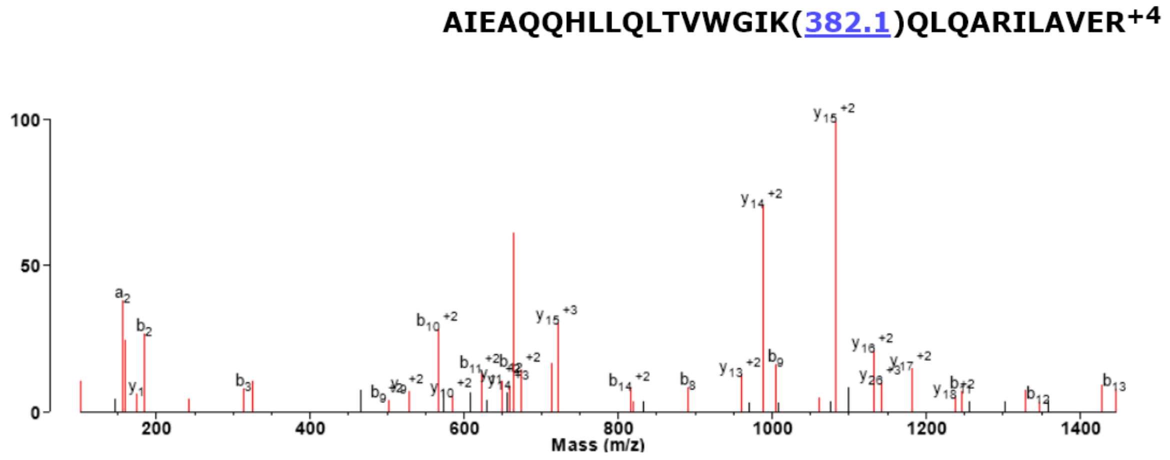

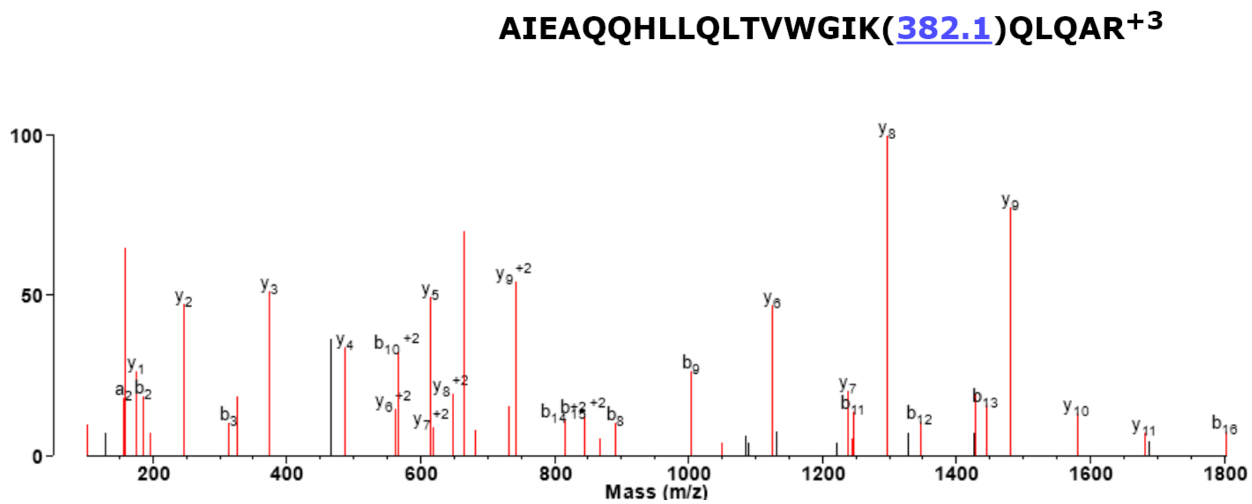

Figure S3. The mass spectrum obtained from the peptides AIEAQQHLLQLTVWGIK(382.1)QLQAR and AIEAQQHLLQLTVWGIK(382.1)QLQARILAVER, confirming the formation of the linkage C23H15FN4O (m/z 382.1224) at K574. The K574 linkage represented > 99% of all modified peptides.

V. Repeat measurements of the compounds in fusion assays

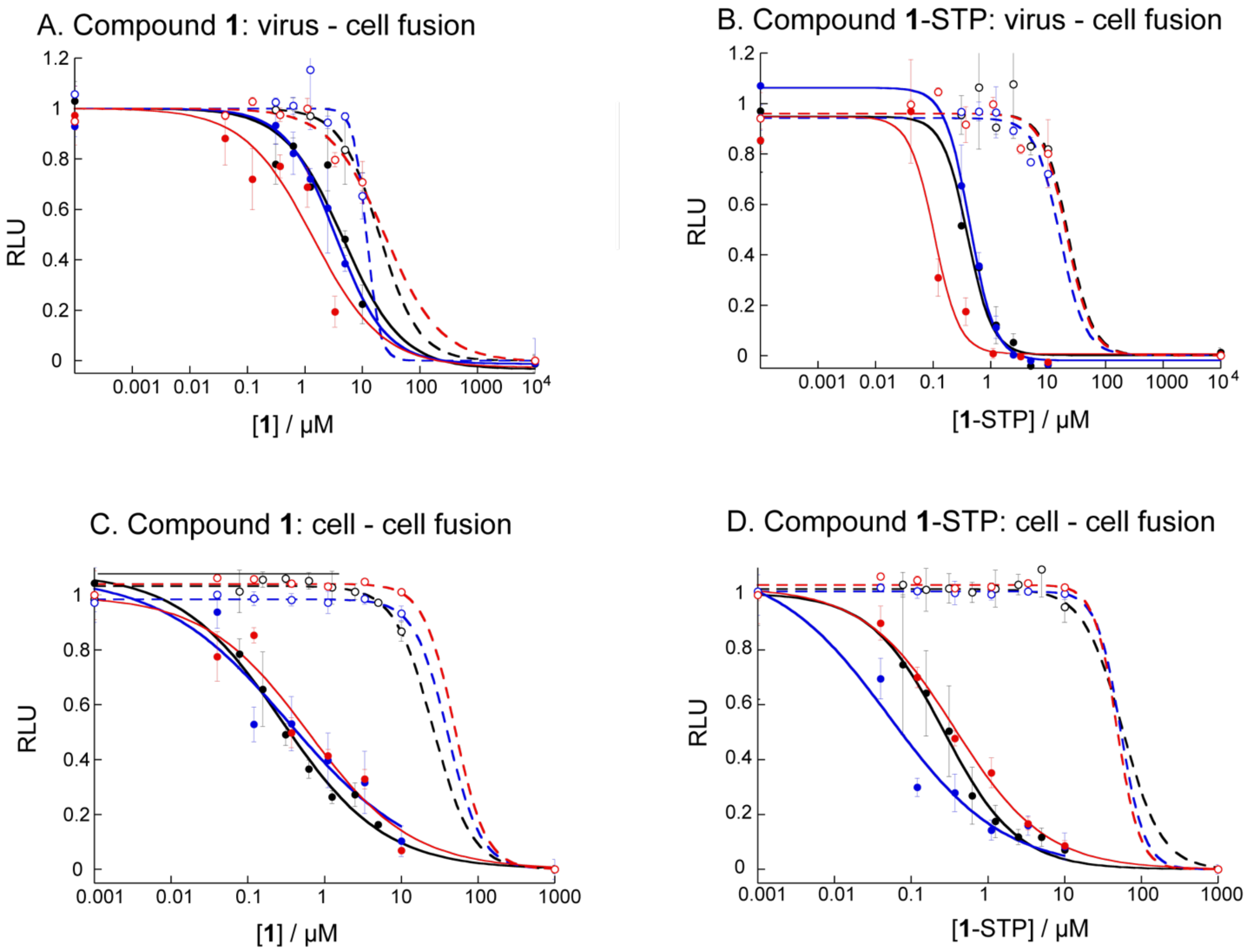

Figure S4.1 Repeat measurements of antiviral activity (A, B) and cell-cell fusion inhibition (C, D) of **1** (A, C) and **1**-STP (B, D). Repeat experiments conducted on different days are shown with black, blue and red symbols and fit to a standard IC_{50} binding curve to give compound efficacy against fusion (EC_{50}) (solid curves) and cytotoxicity (CC_{50}) (dashed curves). Fusion was measured as Relative Luminescence Units and toxicity as Relative Fluorescence Units. The values obtained were used to calculate the entries to Table 2.

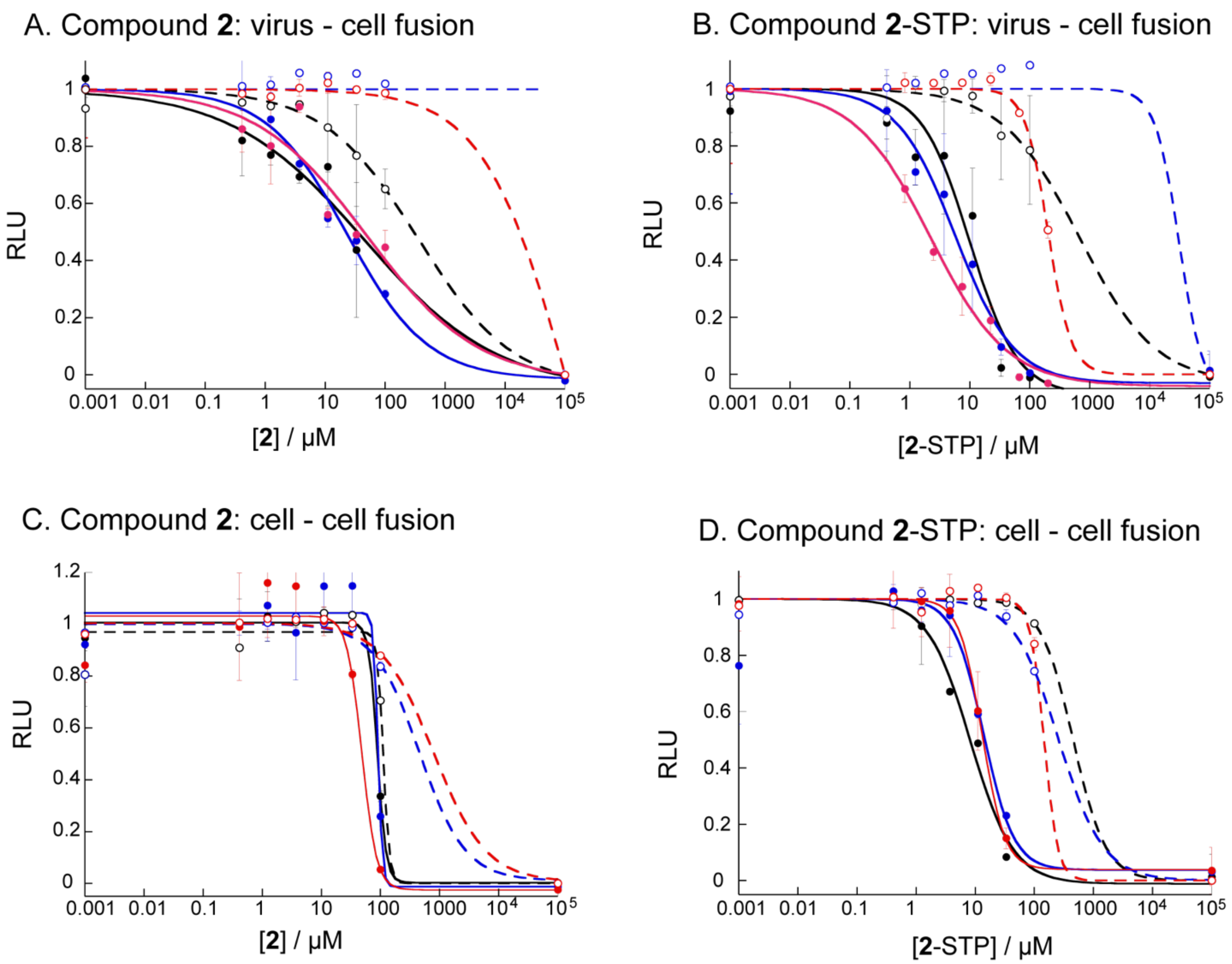

Figure S4.2 Repeat measurements of antiviral activity (A, B) and cell-cell fusion inhibition (C, D) of **2** (A, C) and **2**-STP (B, D). Repeat experiments conducted on different days are shown with black, blue and red symbols and fit to a standard IC_{50} binding curve to give compound efficacy against fusion (EC_{50}) (solid curves) and cytotoxicity (CC_{50}) (dashed curves). Fusion was measured as Relative Luminescence Units and toxicity as Relative Fluorescence Units. The values obtained were used to calculate the entries to Table 2.

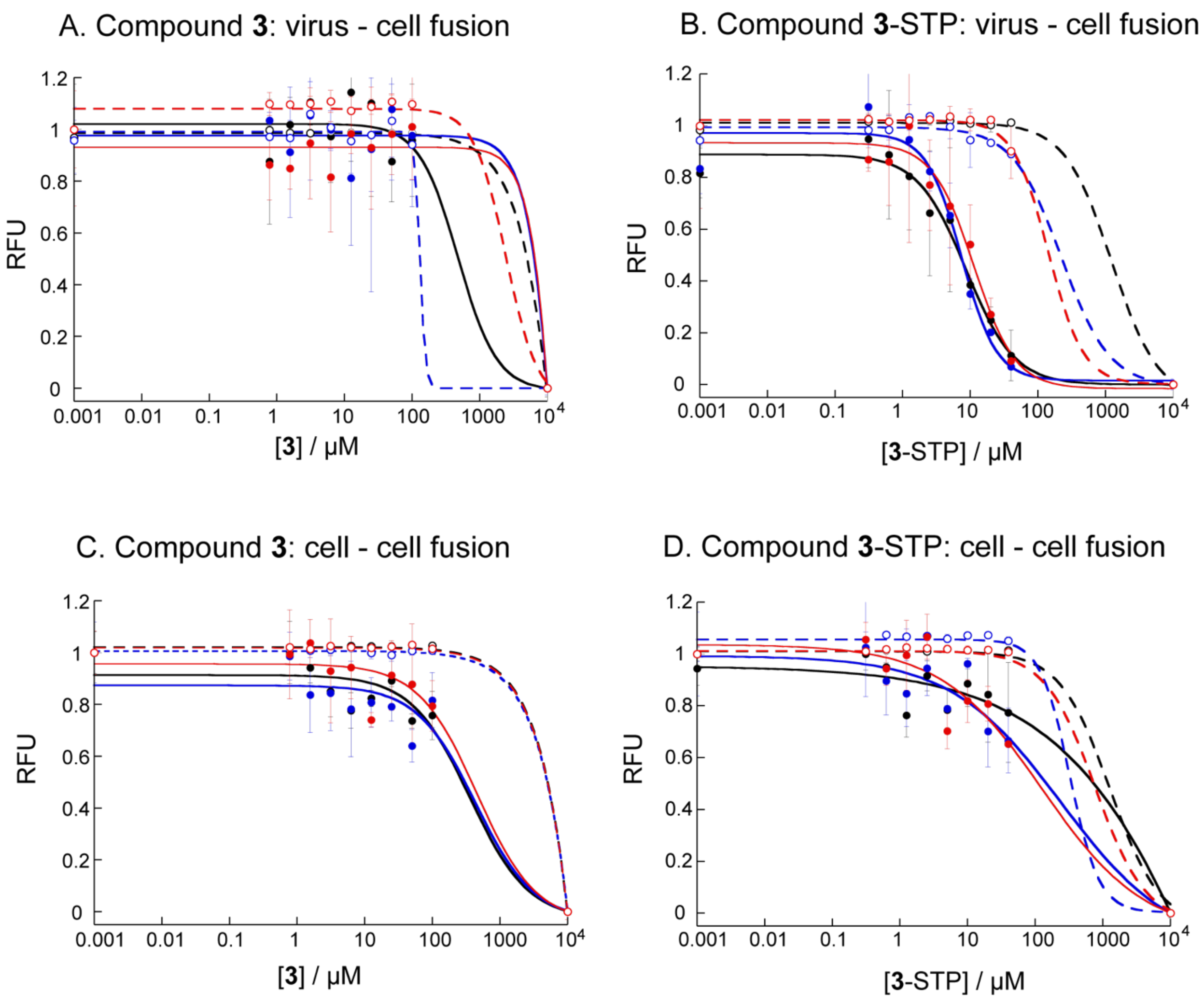

Figure S4.3 Repeat measurements of antiviral activity (A, B) and cell-cell fusion inhibition (C, D) of **3** (A, C) and **3**-STP (B, D). Repeat experiments conducted on different days are shown with black, blue and red symbols and fit to a standard IC_{50} binding curve to give compound efficacy against fusion (EC_{50}) (solid curves) and cytotoxicity (CC_{50}) (dashed curves). Fusion was measured as Relative Luminescence Units and toxicity as Relative Fluorescence Units. The values obtained were used to calculate the entries to Table 2.

References

1. Zhou, G.; Chu, S.; Nemati, A.; Huang, C.; Snyder, B.A.; Ptak, R.G.; Gochin, M. Investigation of the molecular characteristics of bisindole inhibitors as HIV-1 glycoprotein-41 fusion inhibitors. *Eur J Med Chem* **2019**, *161*, 533-542, doi:S0223-5234(18)30918-8 [pii]10.1016/j.ejmech.2018.10.048.
2. Guan, S.; Price, J.C.; Prusiner, S.B.; Ghaemmaghami, S.; Burlingame, A.L. A data processing pipeline for mammalian proteome dynamics studies using stable isotope metabolic labeling. *Mol Cell Proteomics* **2011**, *10*, M111 010728, doi:10.1074/mcp.M111.010728.
